# Supplementary material for: Evaluation of the Hydrophilic/Hydrophobic Balance of 13X Zeolite by Adsorption of Water, Methanol, and Cyclohexane as Pure Vapors or as Mixtures
Source: Nanomaterials (Basel). 2024 Jan 18;14(2):213. doi: 10.3390/nano14020213 (PMC10819054; doi:10.3390/nano14020213)
Supplement: Supplementary file 1 [file nanomaterials-14-00213-s001.zip › nanomaterials-2812364-supplementary.pdf]

Evaluation of the Hydrophilic/Hydrophobic Balance of 13X Zeolite by Adsorption of Water, Methanol, and Cyclohexane as Pure Vapors or as Mixtures

Meryem Saidi [1], François Bihl [2], Olinda Gimello [1], Benoit Louis [2], Anne-Cécile Roger [2], Philippe Trens [1] and Fabrice Salles [1,*]

[a] ICGM, Univ Montpellier, CNRS, ENSCM, Montpellier, France

[b] ICPEES, Univ Strasbourg, CNRS, ECPM, Strasbourg, France

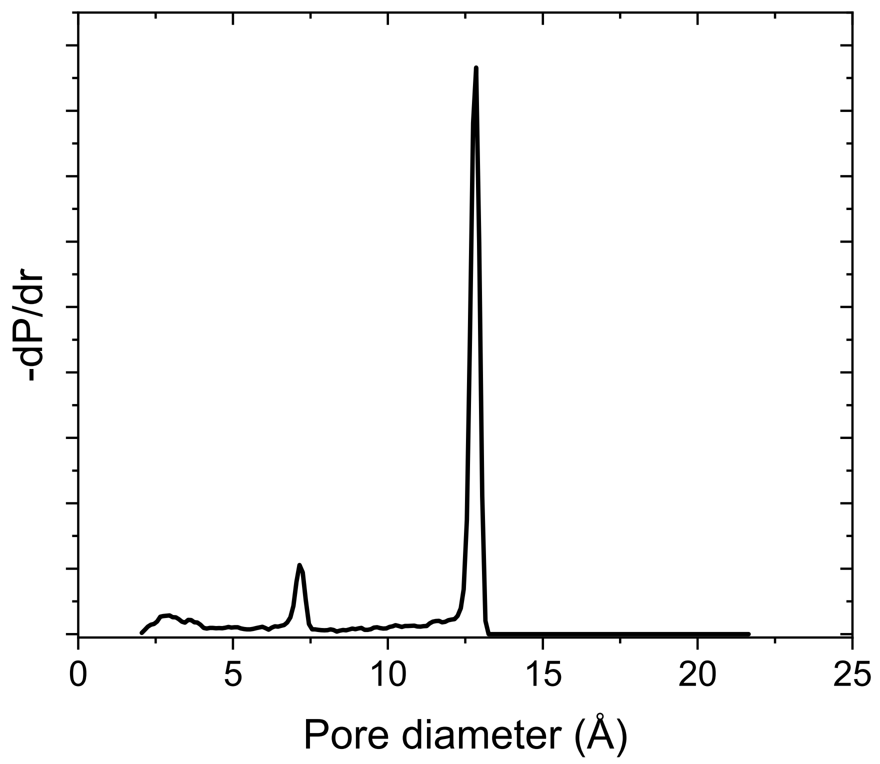

Figure S1. Pore size distribution obtained from the crystal structure using the methodology developed by Gelb and Gubbins (Gelb, L. D.; Gubbins, K. E. Pore Size Distributions in Porous Glasses: A Computer Simulation Study. *Langmuir* **1999**, *15*, 305– 308, DOI: 10.1021/la980841865).

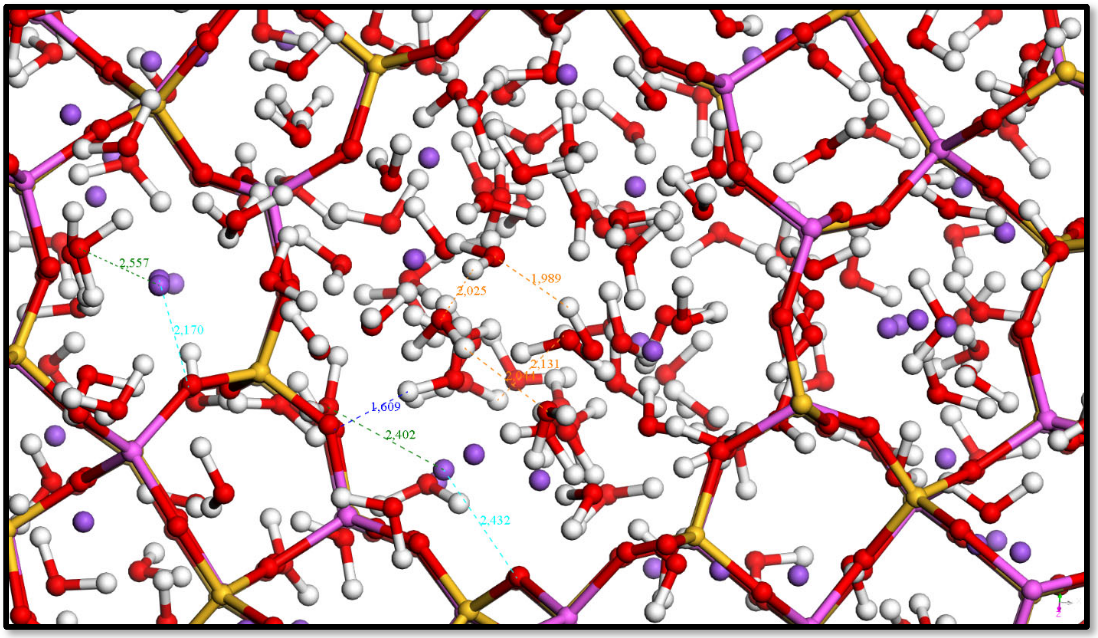
2,557
2,170
2,025
1,989
2,131
1,609
2,402
2,432

(**a**)

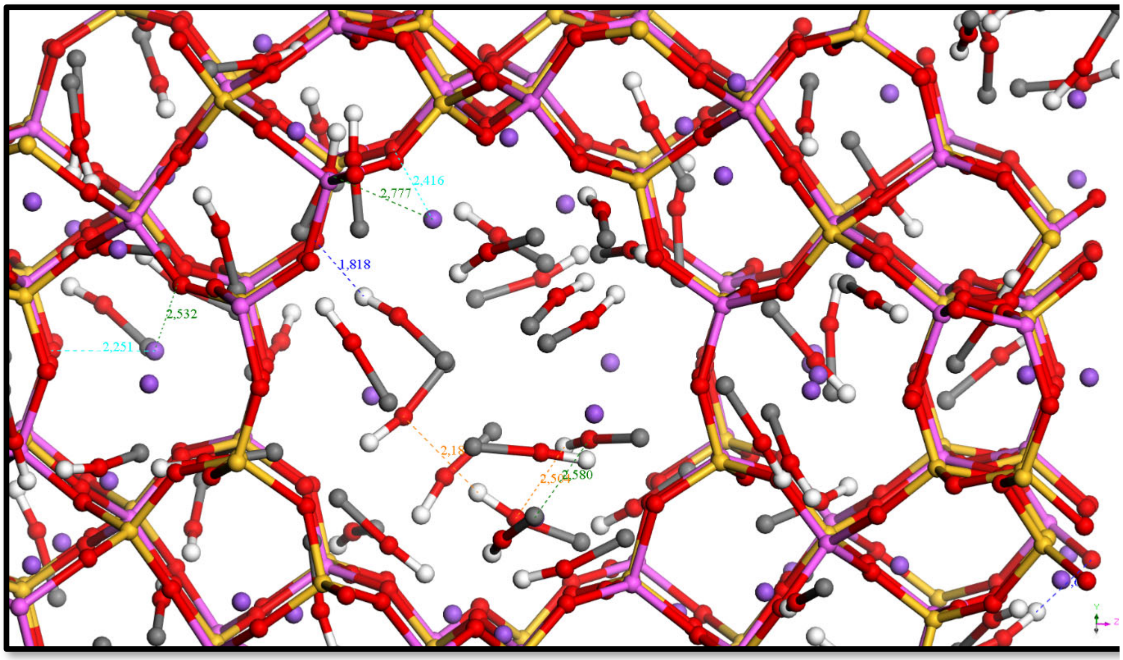
2,416
2,777
1,818
2,532
2,251
2,580

(**b**)

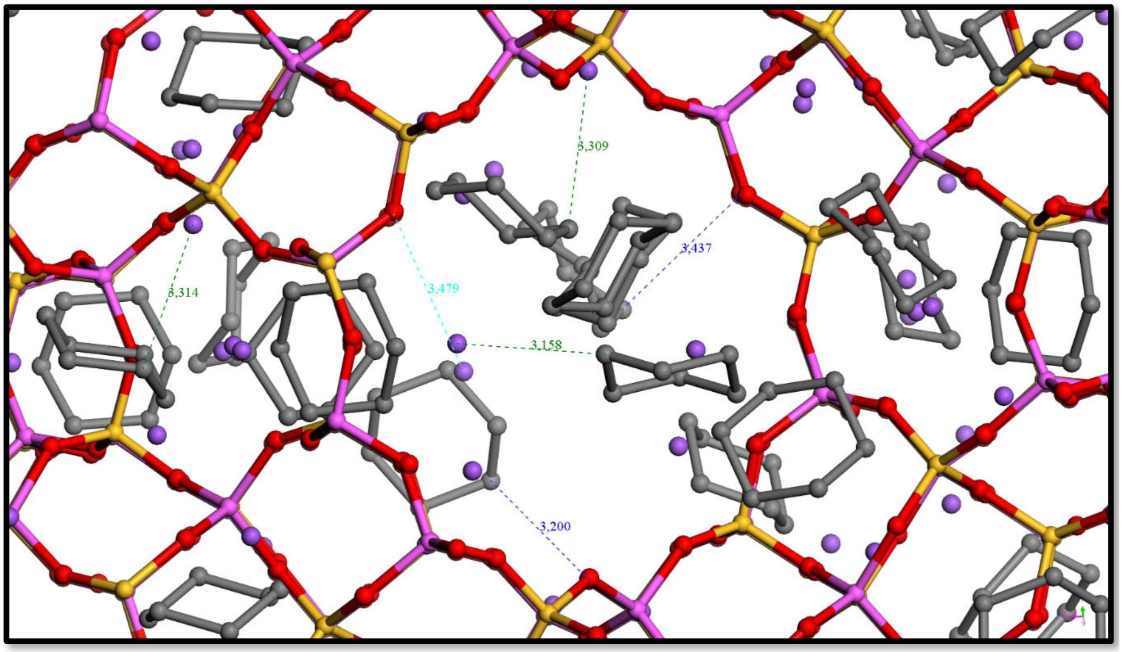

(c)

Figure S2. Snapshots obtained at the saturation of the simulated sorption isotherms at 25°C in the case of (a) water sorption; (b) methanol sorption; (c) cyclohexane sorption.
